# Editorial: Cardiac fat in metabolic and endocrine diseases

**DOI:** 10.3389/fendo.2023.1271565

**Published:** 2023-08-31

**Authors:** Astrid Soghomonian, Bénédicte Gaborit, Federico Carbone, Frédéric Castinetti, Anne Dutour

**Affiliations:** ^1^Aix-Marseille University, INSERM, INRAE, C2VN, Marseille, France; ^2^Endocrinology, Metabolic Diseases and Nutrition Department, AP-HM, Marseille, France; ^3^First Clinic of internal Medicine, Department of Internal Medicine, University of Genova, Genova, Italy; ^4^IRCCS Ospedale Policlinico San Martino, Genova – Italian Cardiovascular Network, Genova, Italy; ^5^Aix-Marseille Université, Institut National de la Santé et de la Recherche Médicale (INSERM), U1251, Marseille Medical Genetics (MMG), Marseille, France; ^6^Department of Endocrinology, Hôpital de la Conception, Centre de Référence des Maladies Rares Hypophysaires HYPO, AP-HM, Marseille, France

**Keywords:** epicardial adipose tissue, myocardial triglyceride content, left ventricular function (LV function), magnetic resonance imaging (MRI), endocrine diseases, metabolic diseases, ectopic fat, myocardial strain

Metabolic diseases, such as obesity and type 2 diabetes (T2D), are major public health concerns, in particular because of their cardiovascular impact. More so than body mass index, adiposity distribution appears to play a key role in obesity-related risk. The expansion and the remodeling of the subcutaneous adipose tissue observed in these pathologies alters the appropriate storage of the energy excess, leading to a phenomenon described as ectopic fat deposition, i.e., the deposit of the triglycerides surplus inside undesirable sites such as the heart (1, 2).

Epicardial adipose tissue (EAT) is a visceral adipose tissue located between the myocardium and the inner layer of the pericardium. EAT is a unique structure with high diverse immune cells and secretome, both implicated in a bidirectional interplay with the myocardium and the coronary vascular wall (3). Myocardial triglyceride content (MTGC) measured in humans using proton magnetic resonance spectroscopy (^1^H-MRS) is a highly flexible source of free fatty acids for the myocardium, notably in young and healthy subjects. The chronic accumulation of triglycerides and ceramides in cardiomyocytes could drive inflammation or fibrosis in patients living with metabolic diseases (4). Previous studies have demonstrated that EAT and MTGC are good predictors of metabolic risk, such as insulin resistance or T2D (5, 6). By their local effects, they can contribute to the pathogenesis of cardiovascular diseases, independently of other visceral fat depots (7, 8).

The present Research Topic entitled “*Cardiac Fat in Metabolic and Endocrine Diseases*” aims to better understand the impact of these cardiac adipose tissues on the heart and the potential ways to modulate cardiac fat.

Due to its special location and its endocrine properties, EAT can communicate directly with the cardiomyocytes and the vascular wall of the coronary arteries (9). Thus, EAT could be implied in the early stages of atherosclerotic plaque formation (10–12). It is also suggested that EAT could be involved in the dysfunction of the coronary microvasculature, an additional mechanism of myocardial ischemia, notably in ischemia with the non-obstructive coronary artery (INOCA). The review by Shan et al. discusses the link between INOCA and EAT and the mechanisms by which EAT can lead to coronary microvascular dysfunction, implicating inflammation, endothelial dysfunction, adipokines regulation (with imbalance between protective (adiponectin or omentin-1) and deleterious adipokines (resistin or leptin) or neurohumoral regulation (notably by activating alpha-adrenergic receptors or the renin-angiotensin-aldosterone system, impairing vasodilation).

MTGC could lead to impaired left ventricular (LV) function and promote the development of heart failure (HF) through lipotoxic mechanisms (13–15). However, human studies are scarce with conflicting results. In order to settle this issue, Soghomonian et al. performed a feature-tracking myocardial strain analysis, a more sensitive marker than conventional parameters to detect subtle changes in LV function in 208 well-phenotyped volunteers and 130 patients living with T2D or obesity. They demonstrate that MTGC could alter cardiac structure, as MTGC was associated with increasing LV mass and may contribute to the development of LV dysfunction, but not in the early preclinical stages of HF. This suggests that MTGC could be seen more as an aggravating factor than a causal one for LV dysfunction in addition to pre-existing deleterious comorbidities.

EAT may exert an effect on cardiac geometry and function by exhibiting local compressive forces and by its paracrine effects on the neighboring myocardium, with the release of pro-inflammatory and fibrotic cytokines (8, 16, 17). Henry et al., in this Research Topic, provide new insights on this topic. The authors performed an elegant study on the kinetic of cardiac geometry after bariatric surgery. In 62 obese patients, weight loss due to bariatric surgery led to a biphasic response in LV geometric remodeling, with an initial reversal of eccentric remodeling followed by a reversal of concentric remodeling. They showed that LV eccentricity index (LVei), a marker of pericardial restraint (a compressive force exerted on the myocardial surface) correlated with baseline EAT volume. They also observed an average EAT reduction of 16% 1030 days after bariatric surgery, which correlated with change in LVei (r=0.43, p=0.0007). This study provides new data on the beneficial role of decreasing EAT volume on cardiac geometry and function.

Therefore, cardiac ectopic adipose tissues appear to be potential targets for reducing the complications of obesity and, notably, cardiovascular risk. Therapeutic interventions, such as pharmacological or surgical interventions and bariatric surgery, can modulate those ectopic fat stores (18–20). Lifestyle changes might also promote cardiac fat reduction, considering that numerous environmental factors are known to be associated with the onset of obesity. In this context, Zhang et al. identified, in a significant cohort of 1151 young adults, that poor sleep patterns, such as evening chronotype and short sleep duration, were associated with an increased waist circumference (WC) and a deleterious metabolic score based on four parameters (BMI, WC, fasting blood glucose, and insulin). Physical activity can also be a cardiac fat deposit modulator. Thapa et al. showed, in 16 women living with obesity, a significant negative correlation between vigorous physical activity levels and cardiac adipose tissue, including EAT and pericardial adipose tissue. This pilot study emphasizes the need to set up large cohorts in order to define adequate exercise, in terms of frequency, intensity, and time, for the modulation of cardiac fat.

Besides, the mechanisms of intramyocardial and intramuscular adipogenesis and lipid accumulation are incompletely understood. Studying intramuscular fat and subcutaneous fat from Laiwu pigs is a good model for this purpose. In this context, Feng et al. analyzed the role of long non-coding RNAs (lncRNAs), miRNAs, and mRNAs in the adipocyte differentiation and lipid metabolism of intramuscular and subcutaneous fat depots of Laiwu pigs. They observed a total of 1209 lncRNAs, 286 miRNAs, and 1597 mRNAs, which were differentially expressed between two types of adipose tissues by deep RNA sequencing. Furthermore, using bioinformatics methods and coexpression network analysis, they showed that the Wnt signaling pathway plays a critical role in intramuscular lipid accumulation in pigs. This study provides a theoretical basis for further understanding the post-transcriptional regulation mechanism of meat quality formation, predicting and treating diseases caused by ectopic fat accumulation.

In summary, this Research Topic highlights the crucial impact of cardiac adipose tissue on the heart in metabolic diseases and provides new insights into the mechanisms leading to abnormal ectopic fat deposition. EAT and MTGC could be potential therapeutic targets in obesity or cardiac care. These interesting data suggest that it is time to include cardiac adipose tissue exploration in routine cardiovascular risk evaluation in patients with obesity or T2D.

## Author contributions

BG: Writing – Review & Editing. AS: Writing. FCar: Supervision. FCas: Supervision. AD: Writing – Review & Editing.
